# Design and Implementation of the Irie Homes Toolbox: A Violence Prevention, Early Childhood, Parenting Program

**DOI:** 10.3389/fpubh.2020.582961

**Published:** 2020-11-16

**Authors:** Taja Francis, Helen Baker-Henningham

**Affiliations:** ^1^Caribbean Institute for Health Research, University of the West Indies, Kingston, Jamaica; ^2^School of Psychology, Bangor University, Bangor, United Kingdom

**Keywords:** violence prevention, parent training, intervention development, early childhood, behavior change, low-and middle-income country

## Abstract

This paper describes the development of the Irie Homes Toolbox, a violence prevention program targeting parents of children aged two to six years. The intervention was designed to complement an existing, teacher-training, violence prevention program, the Irie Classroom Toolbox, thus promoting an integrated approach across home and school settings. The Irie Homes Toolbox was developed through a four-stage process by integrating data from theory, formative research, and practice to ensure the intervention is acceptable, feasible, relevant, and effective in the context. The perspectives of Jamaican preschool teachers and parents of preschool children, who are the end users, were integrated into the design of the intervention throughout the development process. Stage one involved integrating theory and formative research to inform the initial intervention design. Stages two and three involved iterative cycles of design, implementation and evaluation of the intervention content, process of delivery, structure and materials. Stage four involved a further cycle of learning through a process evaluation conducted as part of a cluster-randomized controlled trial. Data from each of these four stages was used to inform the design and ongoing revisions of the toolbox with the aim of developing a low-cost, scalable and sustainable intervention for the Jamaican context. The resulting program is theory-informed and uses empirically derived content and behavior change principles operationalized for the context in which it will be delivered. The Irie Homes Toolbox is suitable for integration into the existing preschool provision in Jamaica, thus utilizing an existing service and existing staff and increasing the likelihood for wide-scale dissemination.

## Introduction

Globally, violence against children exists in almost all countries with the violence often being committed by caregivers (1). This violence is mainly due to the use of harsh discipline practices. The use of psychologically aggressive (e.g., name-calling, yelling) and physically violent (e.g., slapping, beating with an object) discipline practices are considered violence against children or child maltreatment (2). The use of physical violence on children is a direct breach of their human rights and an increasing global concern (3). Approximately 300 million children aged two to four worldwide are disciplined violently by their parents on a regular basis (1). It is estimated that 80% of children globally are spanked or hit at home (4). The use of physical punishment has been shown to be associated with increased child externalizing behavior, antisocial behavior, internalizing problems, lower cognitive ability, and lower self-esteem (5). These associations may also persist into adulthood resulting in increased violent and/or criminal behavior, antisocial behavior, mental health problems, and perpetuation of violence against their own children or spouses (5–7).

Parenting interventions are a key strategy used to prevent violence against children (8). A meta-analysis of child maltreatment parenting programs reported reductions to child maltreatment with an effect size of 0.2 SD, and reported benefits to parents' positive parenting practices, attitude, and self-confidence (9). The majority of studies in the meta-analysis included children younger than five years and interventions were delivered individually (e.g., through home-visiting or in clinic-based sessions), through group parenting sessions or a combination of both. In addition to preventing violence against children, these programs may also prevent child conduct problems, including the development of aggressive behavior (8). This is achieved by educating parents about the importance of positive attention and training them in non-violent alternatives to physical discipline (10).

Jamaican parents regularly use verbal aggression and physical punishment to discipline their children (11). In a study on parenting practices in 24 developing countries, 84% of Jamaican caregivers reported using physical discipline on their children aged five and younger; this was the highest prevalence of all the countries (12). In 2016, Jamaica became one of the first pathfinder countries in the global partnership to end violence against children. Both non-governmental organizations and the government have joined forces to end violence against children (13). The pervasiveness of caregivers' use of violent discipline strategies combined with the push globally and nationally for strategies to end violence against children, show an urgent need for a parenting intervention targeting violence prevention that can be effectively disseminated at scale in the Jamaican setting. Previous qualitative work with parents of preschool children in Jamaica has shown that although parents report frequent use of corporal punishment with their young child, they believe it is undesirable and ineffective suggesting that they would be receptive to training in behavior management (14).

Integrating such programs into existing services would promote parent participation and program sustainability. The most common services accessed by parents of young children are the health and education sector. For example, a parenting program for parents of two to six year old children, implemented in child health clinics in Iran decreased dysfunctional parenting practices and child physical and emotional abuse (15) and a training program for parents of three to seven year old children in Liberia, delivered in school settings, reduced the frequency of harsh punishment, and increased positive parenting practices (16). In Jamaica, we have demonstrated in efficacy trials that integrating parenting interventions into the existing primary health service has the potential to improve child development (17, 18); however sustaining these interventions has proved difficult due to staff workload (19).

Jamaican preschools cater to children aged three to six years and over 98% of young Jamaican children attend preschool. Training Jamaican preschool teachers in appropriate discipline techniques has shown benefits to teachers' child behavior management practices (20). We have developed a teacher-training, violence prevention program, the Irie Classroom Toolbox, to prevent violence against children (21). The Irie Classroom Toolbox has been evaluated with preschool and grade one primary school teachers and has shown large reductions to teachers' use of violence against children (22, 23).

Developing a complementary parent-training program to be implemented in preschools is a logical next step and has the potential for near universal coverage. Integrating the Irie Homes Toolbox into the services offered to parents through community preschools maximizes on this training of preschool teachers as (1) teachers trained in the Irie Classroom Toolbox can deliver the intervention with the parents, and (2) it promotes an integrated approach across home and school settings. In addition, integrating training in violence prevention into early childhood educational services will promote a high quality caregiving environment for young children that is safe, secure, and nurturing, in addition to providing cognitive stimulation. Provision of integrated violence prevention and early childhood development interventions thus promote child development across multiple domains, address multiple risk factors, and help children develop to their full potential (24, 25). Early childhood is a critical period for children's development and optimal development provides the foundation for future physical and mental health (26). Integrated interventions thus have the potential to lead to population-level improvements in child health and well-being (27).

This paper describes the development of the Irie Homes Toolbox, a violence prevention parenting program for parents of children aged two to six years. The main aims of the Irie Homes Toolbox are: (1) to prevent violence against children by parents/caregivers of young children, (2) to promote positive parenting practices including positive discipline, and (3) to prevent the early development of antisocial behavior in young children. The Irie Homes Toolbox was developed as a complementary program to the Irie Classroom Toolbox to promote an integrated approach to child behavior management across home and school settings. It was developed to be integrated into the existing preschool network and to be suitable for implementation by preschool teachers. Preschool teachers have regular contact with parents and are ideally placed to engage parents in a parent-training program being conducted at school. “Irie” is a Jamaican word that describes feeling at peace and in harmony with oneself and with others.

## Methods

**Figure 1:**
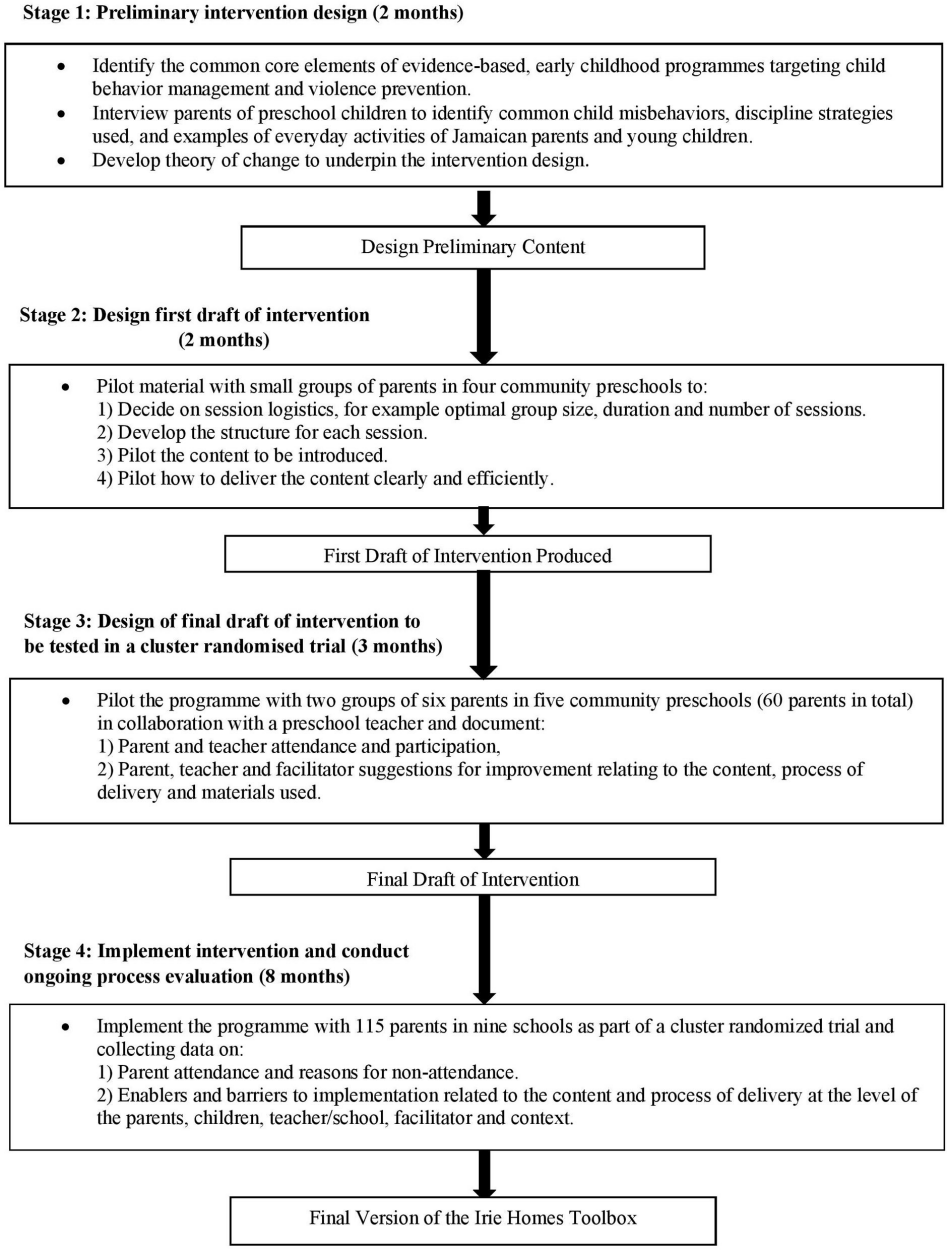
Stages in the development of the Irie Homes Toolbox.

The Irie Homes Toolbox was developed over fifteen months using the framework of the UK Medical Research Council Guidance on Developing and Evaluating Complex Interventions (28). The development process consisted of four stages (Figure 1). The first stage involved integrating theory, empirical evidence, and the perspectives of the end users to inform the initial design of the program; the second stage involved repeated piloting of individual sessions to develop a first draft of the intervention; the third stage was a full piloting phase in which the entire intervention was piloted in five different preschools with groups of six parents, and the fourth stage involved conducting a quantitative and qualitative process evaluation of the intervention as part of a cluster randomized controlled trial. Within and between each stage, ongoing revisions were made to the intervention content, mode of delivery, structure, and materials based parents' and preschool teachers' suggestions and on the observations and experiences documented throughout the process by the research team. These iterative cycles of design, implementation, and evaluation were conducted to promote the acceptability, feasibility, relevance, and effectiveness of the intervention with the target population. All activities in the four stages of the development process were conducted by the authors of the paper and a female research assistant with extensive experience working in Jamaican preschools. Ethical consent for the study was obtained from the University of the West Indies (ECP 144, 17/18) and from the School of Psychology, Bangor University (ref: 2018-16364). Signed, informed consent was obtained from all parents and teachers participating in the study.

### Stage One: Preliminary Intervention Design

#### Methods

In stage one, conducted in December 2017 and January 2018, we integrated evidence from theory, empirical research studies and the perspectives of Jamaican parents. The first step involved identifying the core components of evidence-based parenting programs related to both the content and methods of implementation (29). The focus was on programs targeting parents of children between two and eight years that aimed to improve positive and reduce negative parenting practices, reduce child maltreatment, and/or reduce child behavior problems. We included programs focussing on child maltreatment and on child externalizing behavior, as both types of program include training parents in appropriate discipline techniques. Programs reviewed included the Incredible Years Parenting Program (30), Triple-P Positive Parenting Program (31), Parent Management Training Oregon Model (PMTO) (32), Parent-Child Interaction Therapy (33), Parent Corps (34), and ACT Raising Safe Kids (35) from high-income countries and Sinovuyo (36), Parents Make a Difference (15), and Projecto Paceria (37) from low and middle-income countries (LMIC). We also examined reviews and meta-analyses of the common core components of parenting interventions to identify the components that lead to more effective parenting interventions to prevent and reduce children's externalizing behavior (38–40) and to prevent and treat parents' use of violence against children (41–43). As the parenting intervention is planned as a complementary program to the Irie Classroom Toolbox, we also identified components from the Irie Classroom Toolbox that would be applicable to parents. We created a working draft of possible content and behavior change techniques that would be used in the intervention from these three sources of information, [that is from: (1) the content and method of implementation used in evidence-based parenting programs, (2) qualitative and empirical reviews of common core components from interventions for externalizing behavior and to prevent violence against children, and (3) the core components from the Irie Classroom Toolbox]. To operationalise these core components for the Jamaican cultural context and to inform the design of the intervention materials, we conducted a series of rapid interviews with parents of preschool children to identify common child misbehaviours, discipline strategies used, and examples of everyday activities of Jamaican parents and young children. Through these interviews, we identified the language used by parents to describe children's positive and negative behaviors and words parents use to praise and to chastise children. This information was essential for designing the intervention so that it resonated with the intended particpants and was appropriate for the cultural and economic context. We approached four inner-city preschools to assist us with this initial development. We chose preschools that were located close to the university, were reasonably representative of inner-city preschools in terms of size, staffing, and facilities available, and with whom we had a good working relationship from previous studies. Parents of children attending the school were selected by convenience and a group of ten parents in each school were interviewed. Interviews were conducted on the school compound and lasted approximately fifteen min. A total of thirty-five mothers and five fathers participated in these interviews. The topic guide included: (1) questions about child behavior (e.g., “What are some things that your child does when they are giving trouble?” “What are the main problems you have with your child?”), (2) questions about parent behavior (e.g., “What are some things you say to your child when s/he misbehaves?” “Tell me some of the reasons you slap your child.” “What do you say to your child when s/he is being good?” What instructions do you have to repeat constantly to your child?”), and (3) questions about child activities (e.g., “What game does your child play and what toys does s/he play with?” “What does your child do after school?”). Finally, we developed a theory of change to underpin the intervention development. The theory of change for the Irie Homes Toolbox is similar to that for the Irie Classroom Toolbox and incorporates the COM-B system for understanding behavior which states that for a behavior to occur and be sustained, three factors are required: capability, motivation and opportunity (44). Behavior change techniques to promote these three factors are thus incorporated into the intervention.

#### Results

**Figure 2:**
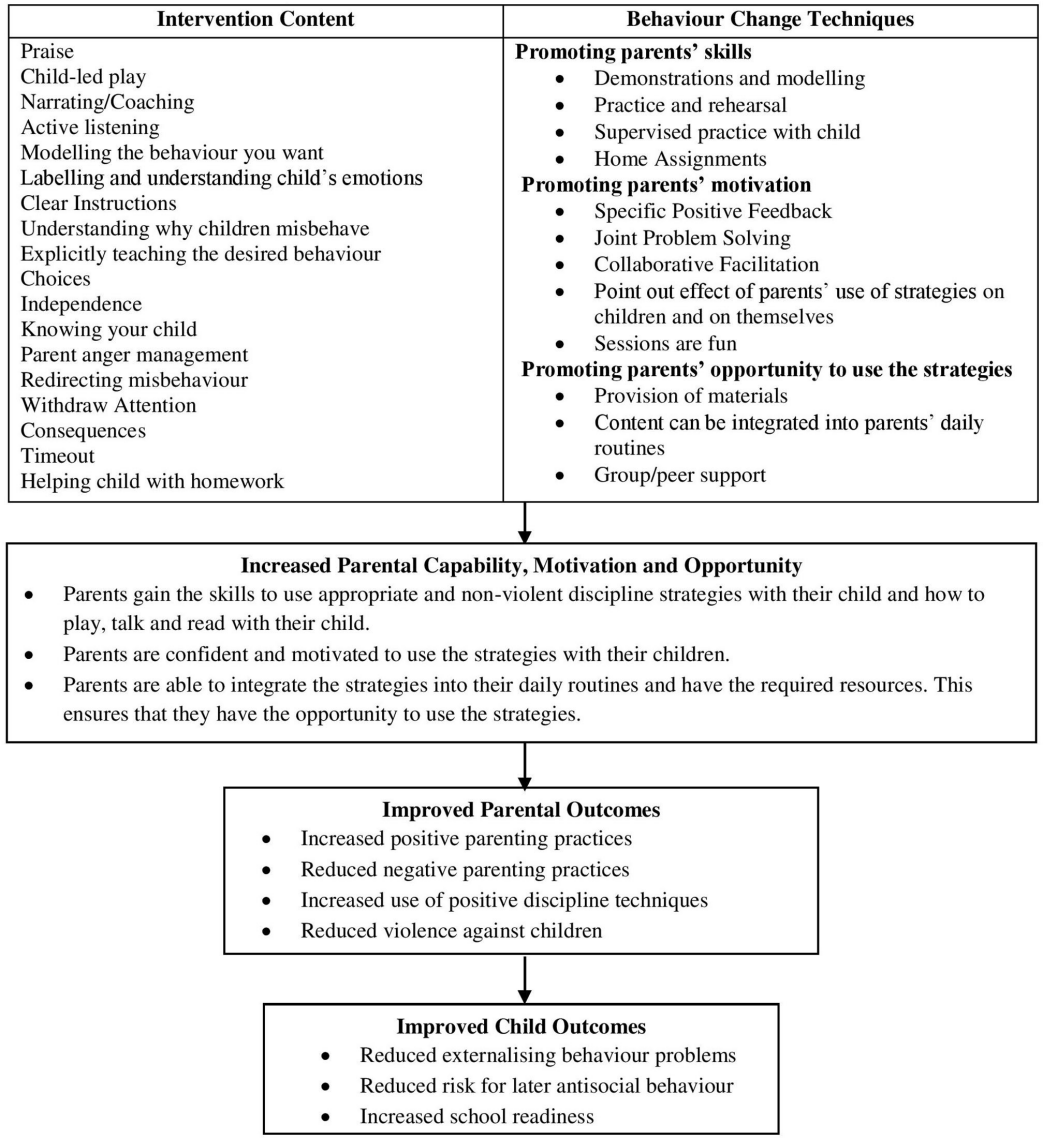
Theory of change.

The common core components of evidence-based parenting interventions and the components of the Irie Classroom Toolbox relevant to parents are shown in Table 1. The core components that have been shown to be most effective in programs targeting child behavior problems vs. child maltreatment share some commonalities and there are also some differences. For programs targeting child externalizing behavior, building a positive parent-child relationship through nurturing, positive and sensitive interactions, the use of praise and positive reinforcement, emotional communication, and learning appropriate discipline methods such as time-out and consequences have been shown to be important components (38, 40). For programs targeting child maltreatment, learning non-violent discipline approaches, daily child-led play, and parental self-management skills have been shown to be most effective (41, 42). In terms of the process of delivering the content, the most effective programs are delivered in a non-stigmatizing way, focus on building parents' self-confidence and involve modeling, rehearsal and practice, supportive feedback, homework assignments, and problem-solving activities (38, 42, 43). These components were thus included in our initial intervention design. We also prioritized relevant content and process components from the Irie Classroom Toolbox as these components (1) have proven effectiveness in the Jamaican setting, and (2) are used by the preschool teachers who will ultimately deliver the intervention to parents. The content used most and most liked by teachers included paying attention to positive behavior, explicitly teaching the expected behavior, giving clear instructions, and interactive reading (21). The training methods most valued by teachers included rehearsal and practice, giving positive and supportive feedback, ensuring teachers recognized the benefits of the intervention to the children and to themselves, group support, provision of necessary materials to implement the intervention, and making sessions fun (21). Table 2 outlines how the parent interview responses were used in intervention development to ensure the program was acceptable, relevant, and feasible for the Jamaican cultural context. The responses were used in the design of role plays, practice activities, visual aids, and problem-solving activities. The theory of change is shown in Figure 2. The core intervention content is delivered using evidence-based behavior change techniques leading to increases in parents' skills, motivation and opportunity to use the strategies with their child. This leads to improved parental outcomes including increased positive parenting, reduced negative parenting, and reduced violence against children. The improvements in parental outcomes in turn lead to improved child behavior and school readiness skills. Using the information from the review of core components, parental interviews and theory of change, we developed an outline of the content and process delivery of the intervention for testing in stage two.

**Table 1:** Core content and behavior change techniques used in parenting interventions to prevent and/or treat child disruptive behavior and child maltreatment and in the Irie Classroom Toolbox.

**Common content in maltreatment and child disruptive behavior programs**	**Content of the Irie Classroom Toolbox appropriate for parents**
Knowledge of child development Parent-child relationship building Child-led play Praise Rewards Promoting children's social skills Giving commands Setting rules Ignoring negative behavior Timeout Consequences Anger management Emotional communication (understanding, identifying and labeling emotions) Parent-self management Consistent responding	Praise Rewards Choices Responsibilities Coaching/narrating Interactive reading Clear Instructions Teaching required skills Emotion regulation Anger management Redirect Withdraw attention Consequences Discipline hierarchy
**Common delivery components in maltreatment and child disruptive behavior programs**	**Behavior change techniques used to deliver the Irie Classroom Toolbox**
Psychoeducation Role-plays, practice and rehearsal Demonstration and modeling Positive and supportive feedback Problem solving Practicing with own child Assigning homework Providing materials Reviewing goals and progress	Modeling Demonstrations Role-plays, practice and rehearsal Positive and supportive feedback Goal setting Collaborative problem-solving Classroom assignments Providing resources Group/peer support

**Table 2:** How parents' interview responses were used in the development of the intervention.

**Topic**	**How the information was used in intervention development**
Common misbehaviours and reasons for slapping child	• Practice activities were designed to demonstrate and practice how to teach desired skills to children (e.g., how to pack up toys, how to put toothpaste on a toothbrush, how to put dirty clothes in the wash basket). • Common misbehaviours were used in a problem-solving activity to encourage parents to consider why children misbehave (e.g., why do children climb the grill, use the mother's lotion, and use bad words). • Role-plays were designed to encourage parents to problem-solve how to use the strategies to manage child misbehavior (e.g., role play of a child having a tantrum, jumping on the bed, child non-compliance). Parents discuss potential solutions and then role play how to implement them. • The information was used to design visual aids depicting children exhibiting common misbehaviours that will resonate with the parents (e.g., jumping on the bed, climbing up the grill, marking on the wall with crayon, hitting their sibling).
Instructions given to child repeatedly	• The phrases reported by parents were used to design role-plays to help parents to give clear instructions. The role plays involved the facilitator using common ineffective instructions (e.g., “Behave yourself,” “settle down,” “relax yourself,” “stop doing that”) and the parents would problem-solve how to change the ineffective to an effective instruction.
Typical games/toys children play with	• The parental responses informed the play materials that would be used in the program. These included coloring, blocks, cars, animals, kitchen set, and picture books. • The responses were also used to help parents think about what activities can be done in Irie Time.
Words used to praise and to reprimand child	• These words were used in the dialogue for role-plays. This ensures the content was relatable to parents.
Household routines, common activities and common issues	• Visual aids were developed to depict everyday routines of children and parents (e.g., eating breakfast, bathing, brushing teeth, getting dressed) and common household chores (e.g., washing clothes, making dumplings, sweeping). These visual aids were used to encourage parents to think about (1) what they can say to their child, (2) what they can praise their child for, (3) what skills they can teach their child, (4) how they can involve their child, and (5) how they can promote choice and independence.

### Stage Two: Designing the First Draft of the Intervention

#### Methods

The aim of stage two was to undertake preliminary piloting of the intervention content and methods of implementation to produce a first draft of the intervention. The activities for stage two were conducted in February and March 2018. We continued to work within the same four community preschools that participated in stage one and convenience samples of five to twelve parents were invited to participate in sessions. The majority of participants were mothers although four fathers and one grandfather were included in the sample. Sessions were conducted at the beginning of the school day. Each individual session was piloted twice with different groups of parents, with ongoing revisions based on lessons learnt during each session. During these sessions one member of the team delivered the content while another team member made notes on: (1) parents' engagement, level of understanding, ability to use the strategies, and what content resonated within the group, (2) ideas for materials and visual aids that would help to promote parents' understanding and engagement, (3) session logistics including session structure, duration and optimal group size. Throughout the process of piloting the sessions, we used the following guidelines to inform our decision making and note taking:

- Be reflective: constantly think of ways to improve the acceptability, feasibility, relevance, and effectiveness of the program.
- Evaluate whether parents understand and are able to use the concepts introduced and plan how their knowledge and skills can be improved.
- If something does not work, or does not work well, problem solve in real-time. Try a different approach and document what happens.
- Pay close attention to the group vibe; if it's flat then something is wrong. Identify ways to maximize participation, fun, and interest.
- Pay attention to individuals in the group, ensure all are participating.
- Make note of scenarios that resonate with the group.
- Note parents' strengths and needs throughout the session.
- Note examples that parents give and use their comments and ideas to revise the content and delivery of the program.

After each session, the notes were discussed among the research team and decisions made to increase the acceptability, feasibility, relevance, and effectiveness of the intervention. These decisions were incorporated into the relevant session prior to piloting the session with a different group of parents.

#### Results

Tables 3, 4 show how we operationalized our key principles of acceptability, feasibility, relevance and effectiveness based on our observations and notes as we piloted the intervention. To promote acceptability, we aimed to: (1) ensure the intervention was fun and engaging, (2) acknowledge parents' current strengths, and (3) help parents see the benefits of the program. The key factors influencing feasibility were considered to be: (1) session logistics, (2) the equipment required to conduct the intervention, (3) whether parents could easily integrate the content into their daily lives, and (4) how to individualize the content to ensure participants' individual needs were met. To promote the relevance of the intervention, we ensured that the material resonated with the parents and met their needs. In addition, as this is a preschool-based program, we included content on how parents can support their child's homework. The acceptability, feasibility, and relevance of the intervention are all critical to its effectiveness. Additional factors considered under effectiveness were: (1) the optimal group size, (2) parent understanding and skill using the strategies, (3) parent motivation, (4) parents' reports on their use of the strategies at home, (5) parents' ability to generalize their use of the strategies, (6) ensuring the sessions are supportive, collaborative and non-critical, and (7) designing the session structure (Table 4).

**Table 3:** Decisions made to improve the acceptability, feasibility, and relevance of the intervention.

**Acceptability**	**Decisions made to increase acceptability**
Fun and engaging	• Session structure incorporates variety to maintain interest. • Sessions start with an action song or game to engage the parents and set the tone for the rest of the session. • Use of games to teach parents how to use strategies. • Skits showing ineffective parenting behaviors are humorous and the behaviors are exaggerated to evoke laughter. • Parents are provided with multiple opportunities to actively participate in the session through games, practice activities, role plays, and discussions. • Visual aids depict funny and relatable scenarios that make parents laugh and prompt thought processes.
Acknowledges parents' strengths	• Parents share positive strategies they are using and are praised for using them. • Parents share how they use the strategies at home with their child. • Parents are encouraged to identify ways in which they are being a good parent and to praise themselves for their positive parenting skills.
Parents see benefits to themselves and their child	• Brainstorms and discussions are done to point out benefits of the strategies. • After practicing a strategy, participants share how using the strategy made them feel and how they think their child will feel.
**Feasibility**	**Decisions made to increase feasibility**
Logistics of session: timing and duration	• The sessions are conducted at a convenient time: either in the mornings when parents drop off their child or in the afternoon after school, depending on the availability of parents in the group. Sessions are conducted while the child is at school so they don't need to arrange for child care. • The sessions are relatively short, lasting about 90 min. This is a realistic amount of time for the teacher to dedicate to the session and appropriate length of time for parents. • The intervention will include eight sessions so the intervention can be feasibly delivered within one school term.
Minimum equipment required to conduct intervention	• All visual aids were designed to be hand-held and we choose not to use a flip-charts and a flip chart stand to display materials or to scribe parents' responses. • No videos, audios or presentations were included in the intervention so no special equipment or electricity supply was required. • To minimize the need for furniture, cardboard placed on parents' laps was used instead of a table for the play activities with their child. • All materials for the program are stored in a portable plastic container.
Parents are able to use strategies in their daily lives	• Content presented easily fits into parents' daily routines with their children. • Resources required are provided or readily available to participants at home. • Strategies are presented and practiced in a clear and detailed way so that parents can easily generalize what is done in the sessions to their real lives. • Parents are supported in setting goals as to how and when they will use strategies in their own lives.
Individualizing the content	• Parents are encouraged to share any difficulties faced with their use of the strategies at home and to engage in group problem-solving.
**Relevance**	**Decisions made to increase relevance**
Scenarios/examples resonate with parents	• Incorporate content that resonated with parents the most. • Content that seemed irrelevant or did not resonate were discarded, reconsidered or refined to make it more relatable. • Additional content and supporting materials were designed based on examples or stories parents shared that resonated with the group.
Include content related to homework	• As the parenting intervention is designed to be integrated into the services provided by preschools, we included a session on how parents can support their child's homework. This included content on homework routines, explaining the task, scaffolding their child, and providing positive and corrective feedback.

**Table 4:** Decisions made to improve the effectiveness of the intervention.

**Effectiveness**	**Decisions made to increase effectiveness**
Logistic of session: group size	• The group size is small (6 participants) so that each participant gets an opportunity to practice skills with individual support. • Groups include an even number of participants to facilitate practicing in pairs as this maximizes engagement.
Parent understanding of skills and using the strategies	• Facilitators model the use of the strategies throughout the sessions. • Facilitators demonstrate clearly what parents are expected to do. • Picture cards depicting the strategies are shown and discussed. • Simple guidelines outlining the steps used in each strategy are drafted to help parents understand and remember the content. • Hand-held charts are used with the steps listed in a clear, succinct manner to reinforce these guidelines. However, the numbers of charts per session were kept to a minimum to ensure sessions didn't become didactic.
Parents are motivated to use the strategies	• Parents are given positive feedback when they use the strategies in the group and when they share how they used the strategies at home. • Benefits of the strategies are discussed. • Parents asked to share how they feel when the strategies are used with them in the role of the ‘child’ and asked how they think their child would feel.
Parents use strategies at home	• Home assignments are given that encourage parents to use the strategies at home. • To add a level of accountability, simple record forms, requiring little reading and writing, were designed for parents to record their use of the strategies at home. • Each session starts with parents sharing how they used the strategies in the previous week. • A take-home card for parents was developed for each session. The card included the key points from the session, written in a succinct, simple, reader-friendly style to act as a prompt for parents to use the strategies.
Parents can generalize to a variety of situations	• Parents set individual goals of how they will use the strategies across different context and situations. This encourages parents to consider how the strategies can be integrated into their daily lives and increases the chances of them using it.
Collaborative, supportive/non-critical	• Parents are encouraged to openly share their struggles with parenting and to engage in group problem-solving. • Parents are encouraged to use their own phrases, expressions, and display their own personality during role-plays. • Facilitator demonstrates the use of the strategy before parents are required to practice. • Facilitator models the use of the strategies throughout the sessions. • Facilitator prompts parents to use strategies. • Facilitator gives parent positive feedback when they use or practice the strategies. • Parents are encouraged to praise each other.
Session structure	• In addition to discussions, demonstrations, practice, and rehearsal with the parents, we decided to include the opportunity for parents to also practice the child-led play activities with their child as an integral part of each session. During this practice activity, parents receive support, and feedback from the facilitators. • Children are brought out for the play session for 15 min and then return to class. Debriefing of the activity and recap of the session then occurs.

A core component of the intervention is to encourage parents to engage in daily child-led play or Irie Time. To ensure parents had the resources available to do this, we decided to include a toy or book in the materials given to parents after each session. We choose toys that facilitated free-play such as blocks, cars, animals and a pretend play set, rather than structured toys such as puzzles, and matching games. We also included three wordless picture books. Two of these books were adapted from the Reach Up and Learn parenting intervention, additional details, and extra pictures were added to make them more suitable for the older child age range targeted in this intervention (45). One book was designed specifically to reinforce content introduced through the program. This book encouraged parents to label children's emotions and included pictures of children expressing different emotions followed by a scenario in which a child may feel that way (for example, a sad face followed by a picture of a child looking sad because they dropped their ice-cream). During the session, we demonstrated the child-led play or book activity, encouraged the parents to practice, and then parents played or read with their child during the session with support from the facilitators. In this way, we capitalized on the fact that the sessions were being conducted at school as children could join the sessions for the play activity only. During the rest of the session, the children were in their classroom and no child-care was required.

By the end of stage two, we had developed an initial draft of the content and process of delivery of the intervention and designed the intervention materials (for example, picture cards, charts, take-home cards for parents, homework assignment record forms for parents, picture books) to be tested in stage three.

### Stage 3: Design of the Final Draft of the Intervention

#### Methods

The first draft of the Irie Homes Toolbox produced through stage two activities consisted of eight 90-min sessions. The full program was then piloted in five new community preschools from April-June 2018. The ultimate goal is for the Irie Homes Toolbox to be delivered by preschool teachers who have been trained in the Irie Classroom Toolbox, hence it was important to include the perspectives of preschool teachers in the development of this parenting intervention. We invited preschools whose teachers had participated in the Irie Classroom Toolbox training to assist us (46). Harsh punishment is common in Jamaica preschools and the rationale for involving teachers who were already familiar with the Irie Classoom Toolbox was that these teachers had developed a good understanding of child behavior management strategies and had experienced the training methods used to help them to change their discipline practices. Six parent-child dyads were recruited from each school, giving a total sample size of thirty parents. The parents were selected based on interest and availability. When a parent dropped out of the program before the sessions began or after attending one session, an additional parent was recruited to participate in the training sessions. The participating parents' children were aged two to six years with a mean age of 3.5 years; 40% of the children were boys. There was one father and the remaining participants were mothers. The preschool principal allocated one teacher to assist the research team and the sessions were delivered in collaboration with a teacher from each school. This ensured that we could include the perspectives of preschool teachers in the intervention design and we could identify challenges associated with teacher participation. Two team members attended each session, one acting as a facilitator and one as a notetaker. After each session, the facilitators and the preschool teacher reflected on (1) parents' level of understanding of the content presented and acquisition of skills, (2) parents' degree of engagement and participation during the session, (3) parents' ability to generalize and apply the skills across multiple activities and contexts, and (4) examples of child and parent behaviors that resonated with the group. These notes were then used to refine the content, delivery, structure, and materials of the intervention with the aim of increasing its acceptability, feasibility, relevance, and effectiveness. These changes were made on an ongoing basis so the refinements could be implemented and piloted in an iterative and cyclical process of design, implementation, and evaluation. In stage three, we also piloted parent recruitment and engagement strategies and made notes on teachers' engagement in the sessions as co-facilitators.

#### Results

**Figure 3:**
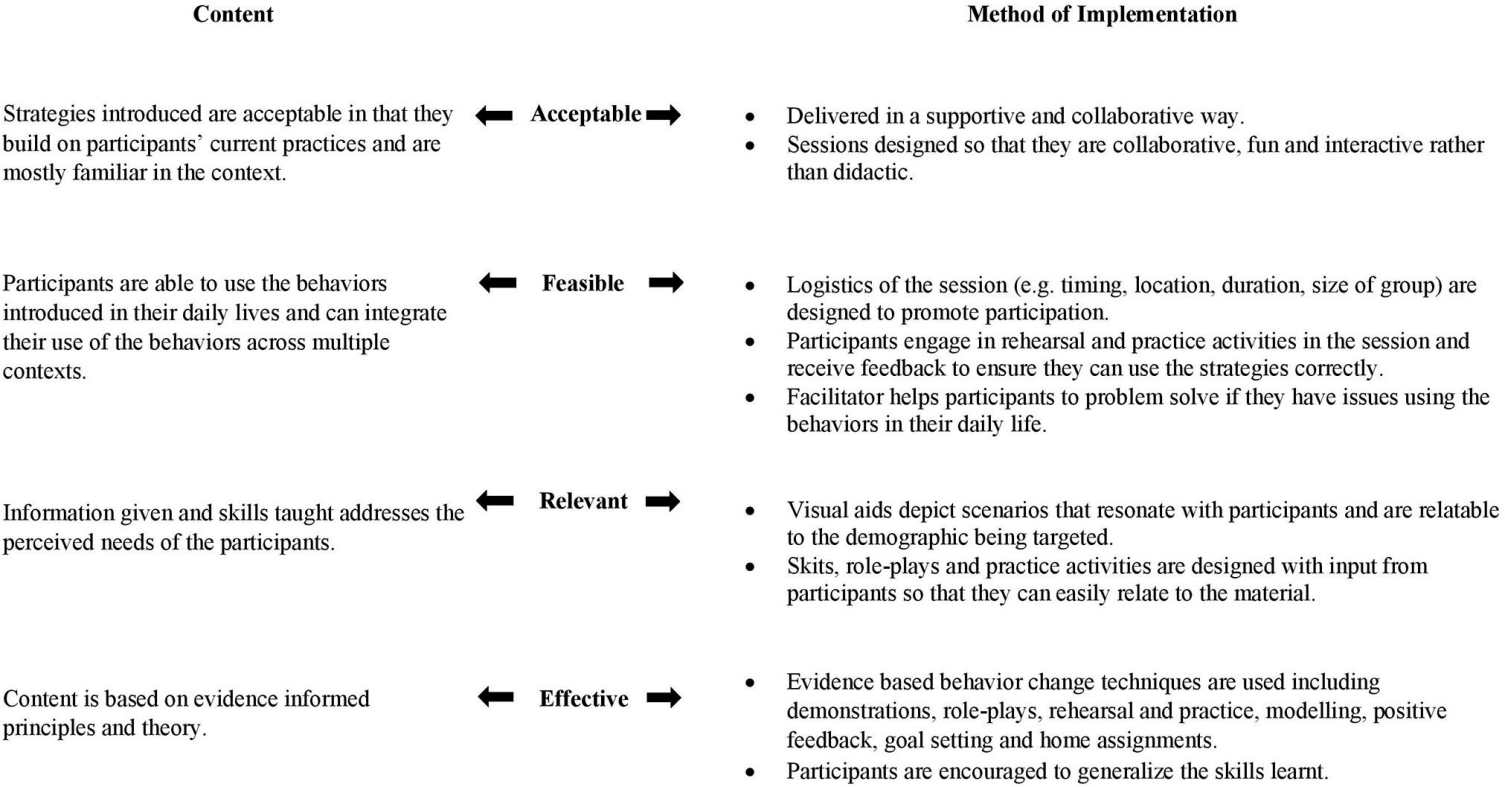
Acceptability, feasibility, relevance and effectiveness of a behavior change intervention.

Table 5 outlines the refinements made to the content, delivery, materials and structure of the intervention based on the data collected from piloting during stage three. Refinements included: (1) adding content related to parents' personal needs, (2) adapting the training methods for content that parents found more difficult, (3) making changes to the session structure, (4) designing additional materials, (5) building more redundancy into the parenting sessions, and (6) providing additional guidelines for the co-facilitator. The data was used to develop the final draft of the Irie Homes Toolbox to be tested in a cluster randomized controlled trial. A summary of how the information from stages one to three was used to ensure the content and methods of implementation used in the Irie Homes Toolbox are acceptable, feasible, relevant, and effective in the context is shown in Figure 3.

**Table 5:** Refinements made to the content, delivery, materials, and structure of the intervention in stage three.

**Type of refinement**	**Examples and rationale for refinement**
Additional content: included content that would explicitly benefit parents	• Self-praise: each week parents share one thing they are proud of as a parent. This was necessary as we recognized that the parents found it difficult to identify and verbalize their positive parenting skills. • Me Time: encouraged parents to take time out of the day to relax and de-stress.
Content misunderstood/hard to grasp: refinement included: changing the delivery format or including an additional mode of delivery to better elucidate the concept.	• Include additional demonstrations by the facilitator prior to having the parents role-play a skill when necessary. This ensured the parents had a better understanding of how to use the strategy prior to participating in the activity. • Prior to participating in a practice activity, show a picture card of a parent using the strategy to stimulate discussion. This helped as parents had more ideas of what they could say or do in a particular activity with their child. • Brainstorm different words, phrases and actions parents can say or do before they participate in a role-play to give parents explicit ideas of how to act in the role-play. This increased their understanding of the content and their confidence to use the strategy.
Session structure: include a structured feedback section in each session where parents share how they completed the home assignment.	• More structure was given to the feedback part of the session to encourage parents to do the home assignments. Each parent shared how they used the strategies at home, using the weekly record sheet as a guide. This demonstrates the importance of completing the assignment and shows that it is expected that program participants engage with the material at home. • If parents were unable to complete the assignment, they are helped to problem-solve to increase the likelihood that they will complete the assignments going forward and are able to use the strategies at jpe.
Additional materials: irie parent oath, irie tower	• At the end of the final session of the program parents sign an oath that they will continue to be an Irie Parent and use the strategies learnt. The oath is designed to help parents self-identify as an Irie Parent. • We designed a visual representation of the program, called the IRIE Tower. The content covered through the parenting sessions is represented by blocks in the Irie Tower. And after each session one or more blocks are added to the tower until it is complete. This helps parents to track their progress and provides the opportunity to continually review prior content. It also acts as a simple memory aid for parents to remember what they have learnt and how to apply the strategies in their daily interactions with their child.
Building in redundancy	• We revised the parent training scripts to ensure that the key concepts are reinforced as often as possible in every session. • The Irie Tower is used every week to review the blocks already covered as well as to discuss the new blocks added during the session.
Additional training guidelines for the co-facilitator	• To promote the engagement of the co-facilitator in the parent sessions, we designed a structured, scripted role in the session for the co-facilitator which was made explicit in the script. This role included participating in role plays and demonstrations, prompting, and coaching parents as they practiced the activities, promoting parent engagement, and assisting parents as they practiced with their child.

The most effective recruitment strategies involved working through the teachers and/or school principal rather than the research team recruiting parents directly. Three of the parents recruited directly by the research team decided they no longer wanted to participate before the program commenced and four parents attended only the first session. These parents were replaced by parents recruited through their child's teacher and/or the school principal. The average attendance of the participating parents was 81% (range 50–100%). We also found that the collaborating teachers would rarely attend the entire session and would leave the sessions frequently to attend to other duties. To promote teacher engagement, we therefore included a structured and scripted co-facilitator role for the teacher in the revised intervention as shown in Table 5.

### Stage 4: Implement Intervention and Conduct Ongoing Process Evaluation

#### Methods

The intervention was tested in a cluster-randomized trial in eighteen community preschools that had not participated in stages one to three. The parenting sessions were implemented during the period September 2018 to April 2019. Preschools were selected to participate in the trial based on the following criteria: (1) had participated in the Irie Classroom Toolbox training and had two or more Irie trained teachers still working at the school, (2) were interested in participating in the program, (3) had parents who consistently dropped children and/or picked up children from school rather than a driver, (4) had no other structured parenting program currently operating, and (5) the principal and all teachers in the school consented to participate in the study. Nine preschools were randomized to participate in the parenting intervention and an ongoing process evaluation was conducted. Parents were recruited at the school by the research team with the help of the teachers and principal. A minimum of six parents were recruited from each school in the Autumn term and an additional six recruited in the Spring term resulting in a minimum total of 12 parents per school. The inclusion criteria for parents were: (1) interested in participating in the program, (2) able to stay back for 90 min either in the morning after dropping off their child or in the afternoon when they came for pick-up one day a week, and (3) parent gave consent for him/herself and his/her child to participate in the study. The quantitative results from the trial will be reported separately. In this paper we are focusing on the process of developing the intervention. One hundred and fifteen parent-child dyads participated in the intervention. The children were aged two to six years old with a mean age of four years; 52.2 % of the sample were boys. Caregivers had mean age of 31 years, 104 (90.4%) were mothers, two (1.7%) were fathers, and nine (7.8%) were other relatives. Less than half the parents completed high school (46%) and 43% were in paid employment. The ultimate aim is for the preschool teachers to implement the intervention. However, in this small-scale efficacy trial we wanted to assess the effectiveness of the program when delivered consistently and with high fidelity; when the intervention is delivered by teachers the quality of implementation may be more variable. Hence, a member of the research team delivered the intervention. Each school principal allocated one preschool teacher to assist the research team by co-facilitating the parenting session. This ensured that we were able to continue integrating the perspectives of the preschool teachers, into the program design. Parenting sessions were held on the school compound (in the school yard or any available room), once a week for eight weeks and lasted 90 min.

The ongoing process evaluation included a quantitative and qualitative component. The quantitative component included recording parent and teacher attendance. For the qualitative component, after each session the facilitators (including the preschool teacher) discussed the session together and then the main facilitator completed a record form that documented enablers and barriers related to: (1) the content of the session (for example, what resonated with parents and what was more difficult), (2) the process of delivery (for example, techniques parents enjoyed the most and least), (3) the context in which the session was delivered (for example, logistics and environment), (4) the involvement of the children, (5) teachers' co-facilitation of the sessions, and (6) facilitators' skills in delivering the session. Notes were also made on individual parents' engagement, understanding, strengths, and needs. Facilitators noted their perceptions of which strategies were most acceptable, feasible, relevant, and effective and suggested solutions to any problems encountered in implementing the intervention. The information from the facilitator record forms was used to further refine the Irie Homes Toolbox.

In addition to the ongoing process evaluation, in-depth, individual, semi-structured interviews were conducted with a subsample of participating parents (*n* = 28) and with one teacher from each preschool who co-facilitated the sessions (*n* = 9). A section of the interview focussed on participants' perspectives of the main benefits of the program and is reported here. One or two parents from each school who had attended a minimum of four sessions were randomly selected for interview. Eighty three (79%) parents had attended four or more sessions and fourteen parents were randomly selected from participants in round one (Autumn term) and a further fourteen parents were randomly selected from participants in round two (Spring term). In-depth interviews with parents were conducted over two phases, within one month after the end of the sessions. Teacher interviews were conducted at the end of round two. Interviews were conducted in a quiet place in each preschool by a female research assistant who had not been involved in implementing the sessions. All interviews were recorded and transcribed. The analysis was conducted using the framework approach which is particularly appropriate for applied research with specific objectives (47). Codes were developed by HBH and a trained research assistant applied the codes to the data and developed the thematic charts. The coding was checked by TF and any discrepancies were discussed and resolved by TF and HBH.

#### Results

Parental attendance averaged 71.5% in round one (Autumn term) and 67.1% in round two (Spring term). 61.8% parents attended six or more sessions with 28.7% all eight sessions. We documented the reasons for parent non-attendance and 79% of the absences were rated as legitimate. Legitimate absences were absences that were deemed unavoidable such as: clinic/doctor visit, called in to work unexpectedly, child being sick, unchangeable appointments, and meeting at older children's school. Teachers co-facilitated a mean of 7.1 (SD 0.9) sessions in round one (the Autumn term) and 3.4 (SD 2.8) sessions in round two (the Spring term). In the Spring term, teachers had many competing activities and responsibilities and attendance was more challenging. In addition, teachers were confident that the facilitator would continue to conduct the sessions if they were absent and this also led to reduced attendance, especially in the face of competing activities.

Facilitators made notes of multiple instances that supported the fact that the intervention developed was acceptable, feasible, relevant, and effective to the participants. In terms of acceptability it was noted that parents would consistently laugh and have fun with the games, role-plays, and visual aids presented. Parents were eager and willing to participate in the different activities during the sessions and they would incorporate their own personality and flair to the activities further confirming their acceptability. The feasibility of the strategies was shown through the parents' reports on how they used the strategies at home in different situations. The relevance of the intervention was illustrated through the discussions in which parents shared instances of issues they were experiencing with their child and would provide examples of how the strategies helped them at home. Throughout the sessions, facilitators documented evidence of effectiveness including parents being able to use strategies with little to no prompting and becoming more confident and skilled in using the strategies as the weeks progressed.

Tables 6, 7 present the evidence from the qualitative interviews on parents' and teachers' perspectives of the benefits of the intervention. Both parents and teachers reported that parents had better emotional self-regulation skills, used less violence against their child(ren) and that parent-child relationships improved. Parents also reported being more confident and proud of their parenting skills, while teachers reported that parents used more praise with their child and showed more interest in school activities. Teachers reported similar benefits to themselves from co-facilitating the sessions, especially in terms of better emotional self-regulation, less violence against children (both in the classroom, and with their own children), and using the other strategies introduced through the program (e.g., praise, modeling, withdrawing attention, and redirecting children's attention) more frequently than previously at school and at home. Parents and teachers also reported similar benefits to children including children being more independent and displaying fewer behavior difficulties (for example, less aggression, and fewer tantrums). Parents also reported increased child compliance and an increase in their child's positive behavior and emotions. Teachers perceived children of participating parents to be more confident in school. Teachers also reported benefits to their relationships with parents and expressed more empathy with and understanding of parents and the difficulties they faced.

**Table 6:** Parents' perceptions of the benefits of the Irie Homes Toolbox.

**Subtheme**	**Examples of quotes**
**Benefits to parents**	
Parent is better able to regulate emotions	• “It help me because I can look at myself and say alright I am going to try to control my anger, I'm going to try to control my temper, I am going to try and control my emotions.” • “I learn how to control my emotions and not to take it out on them. When they are doing anything is either I correct them or give them their time or I send them in their room or something.” • “She teaches us when ignorancy (anger) come we must take in a little breath. and know how to cool down with them because during that time we taking our breath, now you thinking something different.”
Parent bonds more with child	• “It make me spend more time with them and help me to identify when something is wrong with them and it also helps to meet their needs more.” • “Mother daughter time. It help me a lot. I would play with her but not that much but since I come to the irie time, I tend to play with her more often, … we sing together, we do a lot of stuff that irie time has teach me.” • “I used to find myself being busy never used to want sit down with my kids. I used to sit down with them but for like one minute or two but nowadays I find myself that I sit with them all hours playing games or reading or so.”
Parent uses less violence against children	• “I stop beating I just start putting him in the corner. I just used to lick (hit) him for.every little thing and I just stop do that and put him in the corner …so I realize that beatings don't have anything to do with it cause you beat them and them still not going to hear.” • “It help me to not to lick (hit) him. The first time I would say nothing not wrong with a little beating and she tell we other ways we can go about it. It really helpful.”
Increased confidence and pride in parenting skills	• “It help me as a mommy, it make me feel proud of myself as a mother.” • “It make me a better parent.” • “I feel like I am a better person overall—my part playing a mommy, I'm a role model.”
**Benefits to children**	
Increased autonomy and responsibility	• “Yeah because all shoes them now, she put them under the dresser plus her dirty clothes, she put it in the basket without me have to tell her.” • “He is not throwing down his things where they are not supposed to be, he puts them where they are supposed to be.”
Increased participation in chores	• “She sweep out the room, she will spread up the bed. Sweep off the veranda, just to get praise.” • “Yes, she want help me cook, she never normally would want to help me cook.” • “Sometime you don't even have to tell him about sweep. He grab di broom and sweep.”
Increased level of compliance by child	• “If you tell her to sit down she listen, it change her a whole heap because first time me talk to her she doesn't listen.” • “It helped me a lot because certain things I would have to be telling *child's name* like every day and I don't have to be telling him again. Like I told him once and he does it.
Decrease in occurrence of tantrums and/or disruptive behaviors	• “…he is more calmer, he know how to share like it helps, even with the blocks. Instead of jumping up and down and doing things that is not right to get your attention.” • “She don't do the rude things that she used to do like fight or curse.” • “She has become less aggressive.” • “Him wouldn't cry, like him would come and say me can get this please? And if me say no you can't get it, him just go sit down. Him wouldn't just bawl (cry) again.”
Program promotes positive behavior and emotions	• “He would do more good things to get your attention, so he wants to be good cause he wants your attention so you get less trouble and more good.” • “Like him just turn this loving person, everyday … as him come home, him just hug you and kiss you, good evening, good morning- as morning come he is coming to wake you up first to come and hug you and kiss you.” • “She's more happy and contented.”

**Table 7:** Teachers' perceptions of the benefits of the Irie Homes Toolbox.

**Subtheme**	**Examples of quotes**
**Benefits to parents**	
Parents show better emotional self-regulation	• “I realize that … they were better able to manage their emotions so they were calmer and I saw it in how they dealt with the children.”
Parents praise their child more	• “Praising the child more, they actually praise the child a lot more now. • Yes and praising the child for what they've done, focussing on the positive.”
Increased parental interest in school activities	• “You are now seeing the parents having more interest in how their children learn and the things that they do.”
Stronger parent/child relationship	• “Parents are spending more time with the child…interacting and getting to know their child, and know what their child is about.” • “He [father] seems to be much closer to his daughter than usual.”
Parents use less violence against children	• “Instead of shouting at the child, screaming at the child, barking at the child and the licking, once they started learning how to manage the behavior, that gradually went.” • “Well, some parents would've spoken to the children in a very negative manner, they would shout at them, they would call them names and you're seeing a minimal amount of that now.”
**Benefits to children**	
Children have fewer behavior difficulties	• “He [child] has changed because him nuh fling down himself anymore … Him nuh throw down and carry on, no tantrum nuh throw, nuh nothing.” • “He always is very aggressive but now he's calming down a little bit more.”
Children show increased confidence	• “It has built their confidence because like before some of them would've been a little bit more on the reserve side now they're interacting more, they're talking out more”
Children show increased autonomy and responsibility	• “Yeah they are excited, always wanting to do something all when nothing is on the floor they are sweeping. Yeah, they just want to be helpful.” • “They're better able to do some stuff for themselves as well … Alright so where they couldn't lace or tie before, you're seeing more children saying teacher I can tie my shoelace or teacher I can button.”
**Benefits to teacher**	
Teachers have increased emotional self-regulation	• “So it actually show me. how to manage my emotions, do not lose temper, being in control, it actually help me to do that.”
Teacher uses less violence against children at home and at school	• “So interacting with this program it has now rounded me a little bit more on how to effectively parent without administering slaps.” • “I make sure that you're not supposed to shout; IRIE Teachers are not supposed to hit.”
Teacher increased use of other strategies from the Toolbox at home and at school	• “It help me with the praising, the modeling, knowing each child in the classroom, so praising and awarding them like that is one of the biggest things for my class, and withdrawing attention.” • “It also helps me deal with my daughter….I give her the clear instructions. and when she do good things, I praise her.”
**Benefits to parent-teacher relationship**	
Stronger parent-teacher relationships	• “We work together and anything they can come to me and they can talk with me, they can send messages, they write notes. We have a communication.” • “It's easier on me now because they understand some of the things that I have been trying to build with the children so they are better able to help me.”
Teacher shows more empathy toward parents	• “So things that I took for granted that they knew, they really didn't know. It was like an eye opener and it taught me how to be a little more patient and tolerant with them.” • “I'm realizing that some of them really didn't know so my patience level stepped up a notch, my tolerance level stepped up a notch.”

Table 8 shows the barriers faced in implementing the sessions with the changes made to address these barriers. This data was used to further refine the Irie Homes Toolbox to produce the final version of the program.

**Table 8:** Areas of the intervention requiring revision as extracted from the process evaluation tool and changes made in stage four.

**Issues identified**	**Changes made**
A core component of the program is the use of modeling (that is being a role model for your child). The strategy modeling was not explicitly introduced, it was discussed and explained when it came up organically during a session.	• Incorporate content in the intervention so that it is explicitly addressed.
Parents sometimes missed a session and the sessions are cumulative. Therefore, a strategy to ensure parents can catch up is required.	• During the feedback portion of the sessions, prompt parents to demonstrate how they used the strategies at home, instead of simply discussing it. This ensures that parents who have missed a session see a demonstration of the skills.
Games were used to introduce some skills and parents sometimes had difficulty generalizing the skills used in a game to an activity with their child.	• After playing the game, conduct a brainstorm of different things parents can say and do with their child during the activity. • Include a demonstration or role-play to show parents how the skills taught in the game can be applied to their child.
For some of the practice activities, parents needed additional scaffolding before being asked to practice the activity themselves.	• Develop different delivery methods to make the skills clearer. For example, incorporate additional demonstrations, provide clear and simple guidelines of the steps demonstrated, use a visual aid showing the strategy in use, and/or brainstorm with parents what they can say or do prior to asking them to practice the activity.
There were times when parents were less engaged with the material in the sessions.	• Make adaptations to maximize parent participation by revising the delivery mode for the content to make it more fun, engaging, and interactive.
Labeling children's emotions was a relatively new concept for parents and they often reported that they did not use this strategy at home.	• Develop additional practice activities in labeling emotions and include additional emphasis on the rationale for labeling children's emotions so that parents view this as important.
Although the simple and clear guidelines outlining the steps in using a strategy were generally helpful, some parents stuck to the guidelines too rigidly and missed opportunities to expand their interaction with child.	• Facilitators need to make it clear that the guidelines can be used like a recipe. The steps provide the overall structure and parents can add additional steps to it according to their child's interests, needs, and responses.
In the session that included problem-solving activities around managing child misbehavior, parents often chose the same three strategies (clear instruction, chillax, and consequences), rather than using the full range of skills introduced in the program.	• Develop a wider variety of child misbehavior scenarios to ensure there is a good match for each strategy introduced through the program. • Developed additional visual aids depicting these child misbehaviours.
Parents sometimes found it difficult to see things from their child's point of view.	• Provide additional opportunities for parents to play the role of the “child” especially in situations in which they were less empathic. • Incorporate discussions around child's point of view.

### Description of the Irie Homes Toolbox

**Figure 4:**
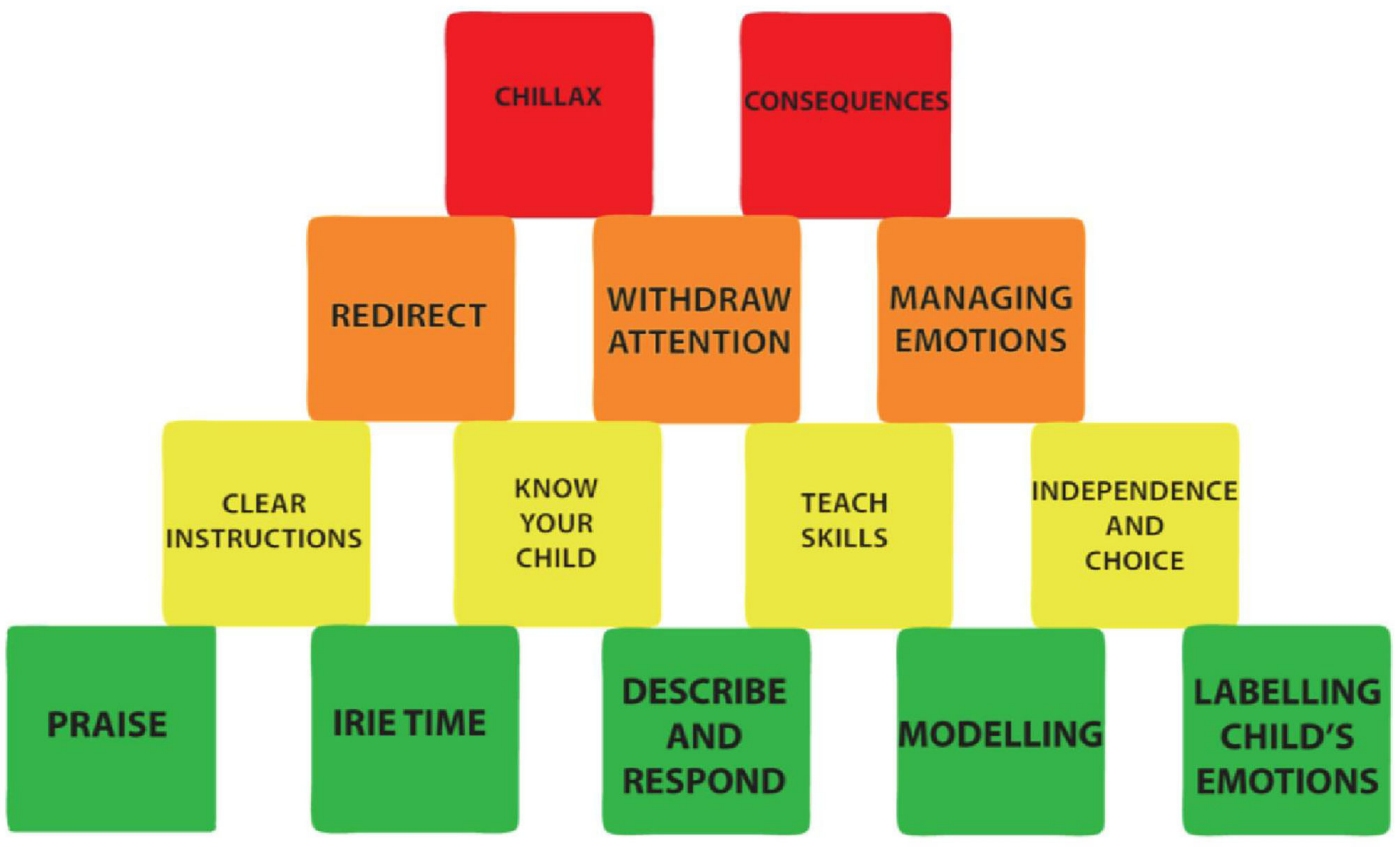
The Irie Tower: pictorial representation of the strategies introduced in the Irie Homes Toolbox. The base of the tower (green blocks) are strategies to promote positive behavior and to build a positive parent-child relationship. The yellow blocks are strategies to prevent child misbehavior. The orange and red blocks are strategies to manage child misbehavior. Parents are encouraged to use the strategies in the two bottom rows liberally and the strategies in the top rows more sparingly.

**Figure 5:**
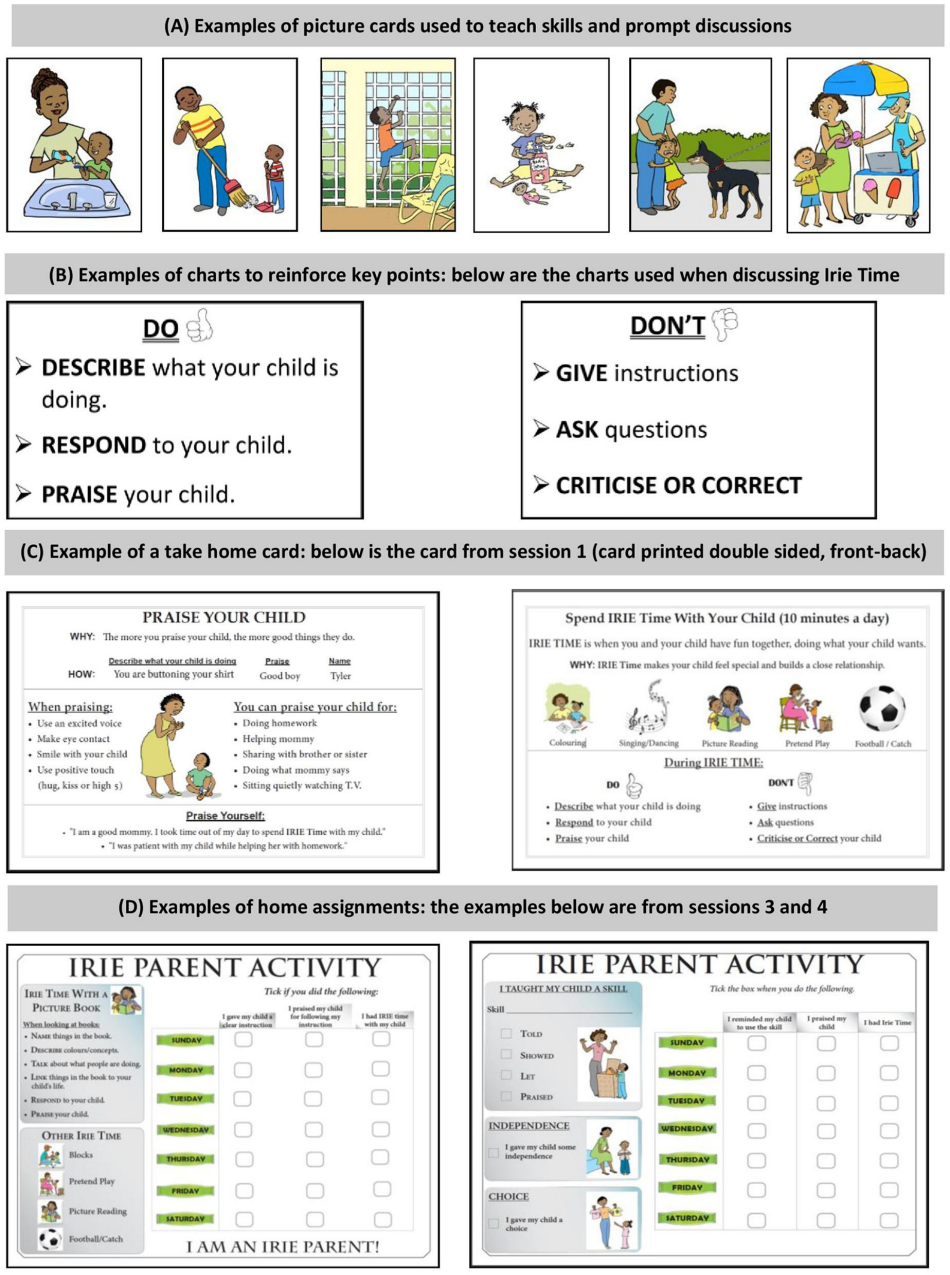
Examples of resources used in the Irie Homes Toolbox.

**Figure 6:**
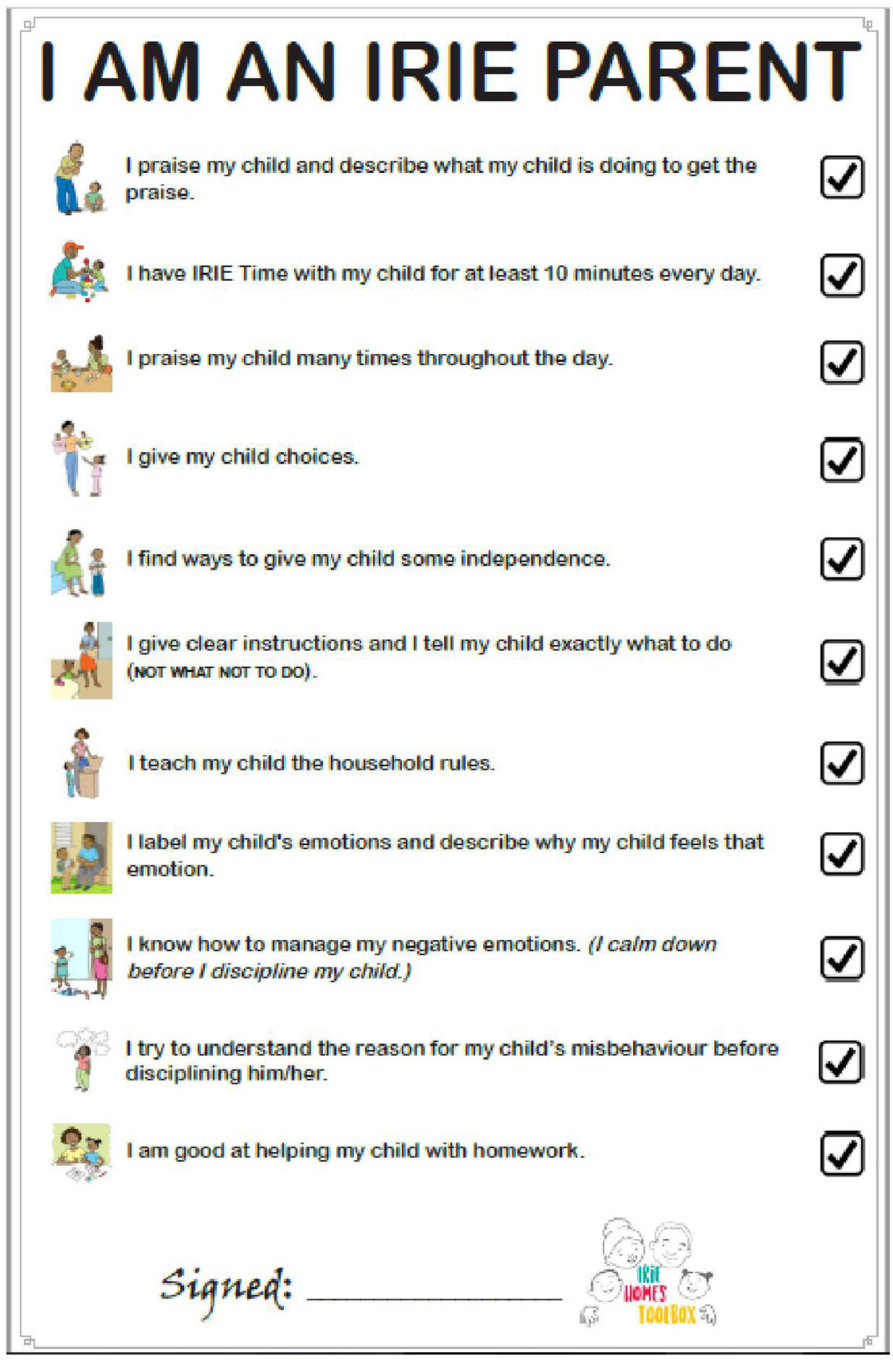
The Irie parent oath.

The intervention developed through the four stages of this study is a violence prevention program for parents of children aged two to six years called the Irie Homes Toolbox. The main goals of the program are to prevent and/or reduce parents' use of violence against children, increase positive parent-child interaction, and prevent and/or reduce child externalizing behavior problems. The Irie Homes Toolbox is a group-based parenting program consisting of eight sessions, each lasting 90 min, delivered once a week for eight weeks. Each of these eight sessions follows a set structure that includes (1) a game or song, (2) feedback from the previous week including a discussion of the home assignment, (3) introduction of a new topic consisting of discussions, role plays, demonstrations, rehearsal, and practice, (4) introduction of a child-led play or picture book reading activity with demonstration, rehearsal, and practice, (5) practicing the child-led play or book activity with their child with supportive feedback, and (6) review, goal setting, and allocating homework assignment. The toolbox consists of content divided into five modules (1) promoting positive behavior, (2) preventing misbehavior, (3) understanding emotions, (4) managing misbehavior, and (5) supporting homework (Table 9). In each session, parents are encouraged to spend daily Irie Time with their child which involves spending quality time with their child and following their child's lead while playing with toys and/or looking at picture books (Table 9). The content covered through the eight sessions of the program is represented in the Irie Tower (Figure 4). The Irie Tower has the blocks representing the positive parenting strategies at the base of the tower as these form the foundation of the program. As the sessions progress, parents are encouraged to use the blocks near the bottom of the tower every day and to only use the blocks at the top when absolutely necessary. Materials used in the Irie Homes Toolbox include materials for facilitators and materials for parents (see Figure 5 for examples of these materials). Materials for facilitators include: (1) a fully scripted training manual including the training scripts for the eight sessions, (2) visual aids (including pictures of parents and children engaged in everyday activities and routines, pictures of parents using the strategies introduced in the program, and pictures of children engaged in common misbehaviors), (3) hand-held charts with key points from the session, and (4) the Irie Tower. Materials for parents include: (1) a take-home card for each session summarizing the content introduced, (2) a homework assignment record for each session, (3) selected toys and picture books for parents to use during Irie Time and (4) an Irie Parent Oath that parents sign on completion of the program (Figure 6).

**Table 9:** Session content of the Irie Homes Toolbox.

**Session**	**Key topic**	**Core content**	**Child-led play activity**
**PROMOTING POSITIVE BEHAVIOR**			
1	Praise	Paying attention to your child's positive behavior. Spending individual time (Irie Time) with your child. Praising yourself.	Coloring
2	Praise throughout the day	Giving your child positive attention during daily activities by describing what your child is doing and responding to your child. Involving your child in household chores. Taking time out of the day to do something you like (Me time).	Blocks
**PREVENTING MISBEHAVIOR**			
3	Clear instructions	How to give your child clear instructions. Knowing your child.	Picture book (My School Day)
4	Why children misbehave and teaching household rules	Understanding the reasons for child misbehavior. Explicitly teaching your child the behavior you want/household rules. Giving your child choice and independence.	Blocks and animal
**UNDERSTANDING EMOTIONS: PARENT AND CHILD**			
5	Emotions	Understanding how your own emotions affect the way you respond to your child's behavior. Ways to calm down when feeling angry. Labeling your child's emotions. Turtle technique to help your child control his/her anger.	Picture book (My Emotions)
**MANAGING MISBEHAVIOR**			
6	Managing misbehavior 1	Redirecting your child's attention and behavior. Withdrawing attention from attention-seeking behaviors. Giving your child positive attention after dealing with a misbehavior.	Blocks, animals, and a vehicle
7	Managing misbehavior 2	How to use Chillax (timeout for misbehavior). Giving appropriate consequences. Giving your child positive attention after using Chillax or other consequences. Problem-solving - applying the strategies learnt to different child behaviors.	Pretend play (kitchen set)
**SUPPORTING HOMEWORK**			
8	Helping with homework	Establishing a homework routine. Scaffolding your child when doing homework. How to give your child positive and corrective feedback.	Picture book (My Day with Mommy)

## Discussion

The development of the Irie Homes Toolbox was guided by theory, evidence, and practice. Through a series of four stages, the intervention content, materials, structure, and process of delivery was tested through an iterative and cyclical process of design, implementation and evaluation with the aim of producing a parenting intervention that was acceptable, feasible, relevant, and effective in the context. The resulting intervention: (1) is theory-informed and incorporates evidence-based content and evidence-based behavior change principles, (2) was developed with participation of Jamaican parents and teachers who are the end users, and (3) was designed to be low-cost and integrated into the existing preschool network thus promoting wider dissemination and sustainability. Evidence from stage four showed that parent attendance and retention in the intervention was good indicating that the intervention was acceptable to parents. Parents and teachers reported benefits to parenting and child behaviors that were targeted through the intervention. Specifically, this included: (1) parents using less violence against children and spending more time building a positive relationship with their child, and (2) children displaying more positive behaviors and fewer behavior difficulties at home and at school. These perceived benefits indicate that parents understood and were willing and able to use the strategies taught through the program, providing evidence for feasibility, relevance, and the perceived effectiveness of the intervention. Parents reported greater confidence in their parenting skills which is likely due to the positive, supportive, and non-critical nature of the program in combination with the changes that they perceived in their own and their children's behavior. The advantages of integrating the intervention into the preschool network were evident as teachers reported benefits to their relationship with the parents and to parents' interest and engagement in school activities. There was also evidence that teachers' involvement in delivering the Irie Homes Toolbox reinforced and enhanced their previous training in the Irie Classroom Toolbox as they reported increased use of appropriate child behavior management strategies in the classroom, in addition to with their own child(ren). The fact that teachers reported benefits to their own personal and professional life is promising as interventions are most likely to be sustained when there are clear benefits to the persons delivering an intervention, in addition to the intended beneficiaries (48). In previous qualitative research with Jamaican preschool teachers, we found no evidence of teachers showing empathy with parents and it was encouraging that in this study, teachers also reported an increased understanding of parents and the difficulties they faced (14). Empirical evidence of effectiveness will be reported separately with the results from the impact evaluation.

There are three broad categories in the literature for the development of behavior change interventions that involve: (1) transporting an existing evidence-based program for use in a new cultural and/or economic context [for example, (49)], (2) developing an intervention specific for the context [for example, (21, 36)], and/or (3) learning while growing which involves continually improving an existing intervention [for example, (50)]. For the development of the Irie Homes Toolbox, we developed a context-specific intervention while also learning while growing through iterative cycles of design, implementation, and evaluation with the results being used to continually improve the intervention. In developing an intervention specifically for the context, we transported and adapted evidence-based principles, rather than transporting an evidence-based program. This approach has several advantages. Firstly, the resultant intervention will be specific to the needs and context of the target group (51) and may lead to a more efficient intervention. For example, in a previous study of training Jamaican preschool teachers in child behavior management, we found that making substantial adaptations and additions to an evidence-based program (in this case the Incredible Years Teacher Training Program) led to a more efficient intervention, requiring less support, and supervision for participants, than when using a minimally adapted version (20). Secondly, there is a growing call for programs to be made freely available globally (52) and this is especially important in low and middle-income countries where the upfront and ongoing costs associated with many packaged programs are prohibitively high. Thirdly, there may also be challenges related to workforce capacity due to the need for highly skilled professionals to deliver some of these existing programs (53). The Irie Homes Toolbox was designed to be integrated into the existing preschool network and to be delivered by preschool teachers, many of whom are paraprofessionals. Fourthly, an intervention that has been developed in the context with participation from local stakeholders is likely to be more acceptable to participants, practitioners, and policy-makers. These factors suggest that transporting evidence-based principles and operationalising those principles for the setting may lead to an intervention that is more scalable and sustainable for that particular context (21).

The Irie Homes Toolbox incorporates the use of evidence-based behavior principles with a particular emphasis on behavior change principles that have been shown to be most valued by Jamaican preschool teachers in a complementary teacher-traning program. These behavior change principles include rehearsal and practice, positive feedback, group support, provision of materials, and ensuring sessions are fun and interactive (21). These methods are used extensively in the implementation of the Irie Homes Toolbox. The use of complementary content and methods of implementation across both the teacher and parent-training programs has several advantages including (1) promoting consistent use of strategies with children across home and school settings, and (2) making it easier for teachers to understand, adopt and implement the program with parents as it mirrors training that they have received. In addition, these interactive and practical training methodologies have been reported to be a key characteristic of effective early childhood parenting programs in LMIC in previous studies (49, 54–56).

The development and/or adaptation of interventions for use in LMICs often entail formative research (14, 57–60). Lachman et al. (36) developed a parenting program to address child maltreatment in South Africa by combining formative research with a theory driven approach that included identifying empirically-derived core components from the literature. To develop the Irie Homes Toolbox, we integrated data from three sources: (1) theory: including behavior change theory, the common core components of parenting programs and the core components used in the complementary teacher-training program, The Irie Classroom Toolbox, (2) formative research: seeking the perspectives of parents and teachers of preschool children, and (3) practice: repeatedly testing and revising the intervention under development with representatives from the beneficiary group. The intense piloting undertaken in this study as part of the intervention design provides the opportunity to pre-test every aspect of the intervention (content, process of delivery, structure, materials, recruitment, and engagement strategies) prior to wider-scale implementation to maximize the likelihood that it will be acceptable, feasible, relevant, and effective in the context. Enablers and barriers to the intervention's effectiveness were documented on an ongoing basis to ensure the intervention can be implemented in the real-world setting. In addition, the intervention was refined iteratively and collaboratively with the end users; incorporating the perspectives of the end users from the initial stages of design onwards increases the acceptability, feasibility, and relevance of the resultant intervention (61). During these learning cycles, we were able to identify problems, implement and evaluate possible solutions, and then implement the learning cycle again with the revised materials. These learning cycles continued until piloting of later drafts of the intervention resulted in little to no changes (29).

Following this development process, we now have an intervention that can be tested in a larger scale effectiveness trial. In the next phase of implementation, the program will be implemented by preschool teachers and supervised by government supervisors from the Early Childhood Commission. There will be a continued need for ongoing learning cycles to identify the strengths and needs of preschool teachers and government supervisors to implement the program and to identify the optimal levels of training and support to ensure the intervention maintains its effectiveness at scale. From a public health stand point, scaling up effective programs is critical to achieving population-level benefits in terms of reduced violence against children and reduced child externalizing behavior problems (62). However, few parenting interventions have been implemented successfully at scale, especially in LMIC, and paying attention to the scalability and effectiveness of an intervention from the design stage onwards is essential to wide-scale dissemination of effective programs. There were several decisions made in the development of the Irie Homes Toolbox to promote future scaling. Firstly, the Irie Homes Toolbox was designed as a complementary program to the Irie Classroom Toolbox. Preschool teachers who have been trained in the Irie Classroom Toolbox, and who are utilizing the strategies taught on a daily basis with children in their class, will deliver the Irie Homes Toolbox to groups of parents within their school. The Irie Classroom Toolbox is currently being disseminated nationally in Jamaica by the Early Childhood Commission, thus providing a strong foundation for the implementation of this intervention. Secondly, the Irie Homes Toolbox was designed to be relatively low cost, requiring few materials, or equipment. We choose not to rely on technology (e.g., video vignettes, audiotapes, presentations requiring a computer, or projector) and designed the sessions so that minimal resources were required. For example, all visual aids are hand-held so no flip-chart stand is required, and we used large pieces of cardboard placed on parents' laps to conduct child play activities rather than use a table. All materials required to implement the intervention are stored in a large, portable, plastic tub. Hence the sessions can be delivered across various locations within the school compound. Thirdly, throughout the development of the Irie Homes Toolbox, we continually revised and refined the intervention content, process of delivery, structure, and materials to promote its acceptability, feasibility, relevance, and effectiveness in the Jamaican early childhood education context. Although the intervention was developed specifically for the Jamaican context, the content, process of delivery, and materials of the program could be adapted to other LMICs. The Irie Homes Toolbox will be made available through a Creative Commons License to the global community.

The methods used in the development of the Irie Homes Toolbox are relevant for use in developing or adapting other educational and public health programs. We recommend an approach that integrates formative research, theory, empirically derived content and behavior change principles, with extensive piloting in the context. Throughout this process, ongoing attention should be given to ensuring the content, process of delivery, and materials used in the intervention meet the four key principles of acceptability, feasibility, relevance, and effectiveness. Figure 3 provides an overview of the key considerations involved in meeting these principles. Furthermore, it is important to engage the end users from the outset and to maintain their engagement throughout the development process. In addition, early consideration needs to be given to how, where, and by whom the intervention will be delivered as that will influence the design of the intervention. For an intervention to be suitable for use at scale, it is likely that it will need to be designed for use by an existing service, existing staff, and using low-cost materials. These factors have a strong effect on all four of the key principles of acceptability, relevance, feasibility, and effectiveness. Conducting a rigorous piloting and feasibility study of a new or adapted intervention prior to implementing an efficacy or effectiveness trial will ensure that the intervention fits the context, will assist in identifying the enablers and barriers to implementation thus maximizing the likelihood of success of the trial and the subsequent uptake of the intervention if is proven effective.

## Data Availability Statement

The datasets generated for this article are not readily available because the data is mostly formative data and is not suitable for sharing as it is difficult to anonymise. Requests about datasets should be directed to h.henningham@bangor.ac.uk.

## Ethics Statement

The studies involving human participants were reviewed and approved by University of the West Indies, Ethics Committee and School of Psychology, Bangor University Ethics Committee. Written informed consent to participate in the study was provided by participating parents and teachers.

## Author Contributions

HB-H and TF contributed to the conceptualization of the study, funding acquisition, project administration, investigation, data curation, data analysis, writing the original draft, and reviewing and editing the manuscript.

## Conflict of Interest

The authors declare that the research was conducted in the absence of any commercial or financial relationships that could be construed as a potential conflict of interest.
