# Corrigendum: Design and Implementation of the Irie Homes Toolbox: A Violence Prevention, Early Childhood, Parenting Program

**DOI:** 10.3389/fpubh.2020.630819

**Published:** 2021-01-13

**Authors:** Taja Francis, Helen Baker-Henningham

**Affiliations:** ^1^Caribbean Institute for Health Research, University of the West Indies, Kingston, Jamaica; ^2^School of Psychology, Bangor University, Bangor, United Kingdom

**Keywords:** violence prevention, parent training, intervention development, early childhood, behavior change, low-and middle-income country

In the original article, there was a mistake in Table 9 as published. Two rows are missing in the published article: the row with information relating to session five, and the row with the subheading for sessions six and seven (Managing Misbehavior). The corrected Table 9 appears below.

**Table 9:** Session content of the Irie Homes Toolbox.

**Session**	**Key topic**	**Core content**	**Child-led play activity**
**PROMOTING POSITIVE BEHAVIOR**			
1	Praise	Paying attention to your child's positive behavior. Spending individual time (Irie Time) with your child. Praising yourself.	Coloring
2	Praise throughout the day	Giving your child positive attention during daily activities by describing what your child is doing and responding to your child. Involving your child in household chores. Taking time out of the day to do something you like (Me time).	Blocks
**PREVENTING MISBEHAVIOR**			
3	Clear instructions	How to give your child clear instructions. Knowing your child.	Picture book (My School Day)
4	Why children misbehave and teaching household rules	Understanding the reasons for child misbehavior. Explicitly teaching your child the behavior you want/household rules. Giving your child choice and independence.	Blocks and animal
**UNDERSTANDING EMOTIONS: PARENT AND CHILD**			
5	Emotions	Understanding how your own emotions affect the way you respond to your child's behavior. Ways to calm down when feeling angry. Labeling your child's emotions. Turtle technique to help your child control his/her anger.	Picture book (My Emotions)
**MANAGING MISBEHAVIOR**			
6	Managing misbehavior 1	Redirecting your child's attention and behavior. Withdrawing attention from attention-seeking behaviors. Giving your child positive attention after dealing with a misbehavior.	Blocks, animals, and a vehicle
7	Managing misbehavior 2	How to use Chillax (timeout for misbehavior). Giving appropriate consequences. Giving your child positive attention after using Chillax or other consequences. Problem-solving - applying the strategies learnt to different child behaviors.	Pretend play (kitchen set)
**SUPPORTING HOMEWORK**			
8	Helping with homework	Establishing a homework routine. Scaffolding your child when doing homework. How to give your child positive and corrective feedback.	Picture book (My Day with Mommy)

The authors apologize for this error and state that this does not change the scientific conclusions of the article in any way. The original article has been updated.

